# Supplementary material for: Intra-operative autologous blood donation for cardiovascular surgeries in Japan: A retrospective cohort study
Source: PLoS One. 2021 Mar 10;16(3):e0247282. doi: 10.1371/journal.pone.0247282 (PMC7946193; doi:10.1371/journal.pone.0247282)
Supplement: Attachment — Submitted filename: Responce_to_Reviwers.docx [file pone.0247282.s001.docx]

Journal requirements:

1. PLOS ONE's style requirements
   **Response**
   I corrected to match.
2. Data Availability statement
   **Response**

I added the follow information in revised cover letter.
“The data generated and analyzed during this study cannot be shared publicly, due to Ethical Guidelines for Medical and Health Research Involving Human Subjects of the Ministry of Health, Labour and Welfare, Japan (a provisional translation is available from [https://www.mhlw.go.jp/file/06-Seisakujouhou-10600000-
Daijinkanboukouseikagakuka/0000080278.pdf](https://www.mhlw.go.jp/file/06-Seisakujouhou-10600000-Daijinkanboukouseikagakuka/0000080278.pdf)). However, other researchers may send data access requests to the Ethics Committee, Graduate School of Medicine, Kyoto University ([ethcom@kuhp.kyoto-u.ac.jp](mailto:ethcom@kuhp.kyoto-u.ac.jp))”

3. Title
   **Response**
   I corrected to match.
4. the date on which the author accessed the databases
   **Response**
   “We accessed the database and got the information of eligible patients in July 2020.” (Lines 88-90)

Reviewer #1:

**Edits by line number:**
**# 52-53: please clarify syntax. Presumably minimizing transfusion is also important for surgeons and patients, too.**

**Response**
 “In CVS, which consumes large amounts of donated blood, efficient transfusion can ensure the proper use of limited medical resources and improve patients’ outcomes” (Lines 53-54)
**# 58: syntax needs to be clarified**

**Response**
“Although the evidence level of IAD was IIB [12], which was not high, the Ministry of Health, Labour and Welfare of Japan approved the procedure in 2016, adding to the universal benefit scheme” (Lines 58-60).
**# 116-117: please revise the sentence for clarity -- presuming that heparin and antiplatelet agents were stopped in the usual fashion**

**Response**
 We changed the term “heparin use” to “bridging anticoagulation ” (Line 118)

As the reviewer pointed out, heparin and antiplatelet agents was stopped in usual fashion, so I deleted the phrase about the cessation of those drugs and added the above phrase, “bridging anticoagulation”.
**# 118-120: Standard accepted definitions for hypothermia should be followed. Deep is <24C, Moderate 24-32C, mild >32C. (Leshnower et al, 2015 Annals Thoracic Surgery)**

**Response**
 “We also considered the interval of intra-operative CPB by the temperature, deep and moderate hypothermia (<32℃), and mild hypothermia (≥32℃)” (Lines 120-122)
**# 150: please edit "arterial" to read "aortic"**

**Response**
As advised, we changed “arterial” to “aortic” (Line 153)
**# 166-167: please revise for clarity.**

**Response**
“The total amounts of transfused RBCs, FFPs, and PLTs during hospitalization are summarized by surgical type in Fig 2. In the IAD group, the amounts of all transfusions in cardiac surgery and FFPs in aortic surgery were smaller than those in the control group” (Lines 166-169).

**# Discussion section is not page numbered. There are multiple areas in the discussion that need copy editing for clarity. When you state that IAD was only approved in 2016 -- approved by whom? The discussion touches on perceived shortcomings of other peer-reviewed work. Please revise to focus instead on what the present study demonstrates.**
**Response**

We have ensured that the page numbering in the discussion section is clear. Besides, we have proofread the text and ensured that the discussion section focuses on the current study results.

**Reviewer #2: 
1. Please ask English native speaker to review and check the manuscript.**

**Response**

Our manuscript was proofread again by an English native speaker. We hope the language of our manuscript now meets your publication standards. We would be happy to make further changes if needed.
**2. Title of the front page has to be same as the title of the manuscript. Do not exclude “in Japan” in the title.**

**Response**

As advised, we have included “Japan” in the title.
**Intra-operative autologous blood donation for cardiovascular surgeries in Japan: a retrospective cohort study**
**3. What is “control group”? I guess it is the patient group without IAD, but you have to mention it.**

**Response**

We have defined the control group as follows:
 “The intervention group included patients who received IAD, and the control group included those who did not receive IAD.” (lines 98-100)
**4. Why did you exclude the cases with CPB time less than 40 minutes? I don’t know what you mean by “indicating possible intra-operative challenges (<40min)”? Why not 30 minutes, why 40 minutes?**

**Response**
In some cases of a short duration of CPB, there is a possibility of unexpected problems, such as input errors or anaphylaxis. With the limited data from the retrospective study, it was difficult to determine an accurate cutoff time. Therefore, we referred to a recent single-center study that excluded cases of less than 40 minutes for the same reason. [Zimmermann E, Zhu R, Ogami T, Lamonica A, Petrie JA 3rd, Mack C, et al. Intraoperative autologous blood donation leads to fewer transfusion in cardiac surgery. Ann Thorac Surg. 2019;108: 1738-1744.]
**5. Did you include the emergency cases? These tend to have more transfusion in general.**

**Response**
We selected scheduled surgeries only. Therefore, we did not include urgent or emergency surgeries.
**6. Did you include off-pump CABG? These tend to have less transfusion in general.**

**Response**
By focusing on surgeries in which cardiopulmonary bypass was used for more than 40 minutes, cases of off-pump CABG were excluded.　
**7. Why do you think “Re-operative for bleeding” of the IAD group is higher than control group in aortic surgery patients? IAD group receive less transfusion, but higher “re-operation for bleeding”? I know it is not statistically significant, but please let me know your opinion.**
 **Response**

Since the findings are based on matching analysis, the calculated “Re-operation for bleeding” varies slightly depending on the selected population. In the new analysis (excluding the preoperative autologous blood group), “Re-operation for bleeding" was slightly more common in the control group, although the difference was not statistically significant. These results may be so because we excluded patients who had received preoperative autologous blood donation.

The higher number of re-operative patients in the IAD group may be due to (1) quantity and (2) quality of coagulation factors.

This is just conjecture, but the possible reasons for the higher number of surgeries in the IAD group are as follows:

(1) Since autologous blood is used, patients with IAD may be less aware of the need to replenish coagulation factors, which play an important role in hemostasis in vascular surgery. It cannot be denied that the intention to save blood transfusion as much as possible may have some impact when using IAD.

(2) The quality of clotting factors in IAD is excellent, as mentioned in the manuscript; therefore, bleeding problems may not be expected. However, if there is insufficient agitation during blood collection, the quality of the clotting factors provided at the time of administration may be lower than expected.

**8. Some control group patients received “PAD”. Do you really think these patients can be in the control group? It is still the autologous blood donation, so PAD may contribute to decrease the transfusion rate, so I think these patients should be considered separately, or excluded.**

**Response**
In this study, we calculated the propensity score by including preoperative autologous blood donation (PAD) as a variable that affects the intention to use IAD and the patients’ outcome. The propensity score was well-balanced (both in terms of the ratio of the number of people using it in the target population and the amount of blood used per person).

However, as the reviewer said, it would be appropriate to exclude patients who received PAD, given the purpose of the study (to examine the use of stored blood and side effects). Therefore, patients who received PAD were excluded from the analysis. However, we obtained similar results.

PAD has rarely been examined in studies on IAD for cardiovascular surgery. This is probably because only IAD is used in other countries, but fortunately, PAD is used in Japan, although the number is small. Therefore, another study should be performed to compare IAD with PAD.

**9. What do you think is the “unmeasured confounding bias”? Please mention the examples.**

**Response**
Coagulation tests: Depending on the case, the coagulation system may be assessed in more detail with ROTEM before the introduction or withdrawal of the artificial heart–lung. The result may affect the intention to use blood transfusion.

Amount of blood loss: Although this factor affects the likelihood of performing blood transfusion, it does not have a large impact on clinical practice because not all blood loss can be measured in clinical practice (collection in the artificial heart–lung or splattering on the floor), and there are few studies that take into account the amount of blood loss.

More detailed information on CPB: Hemoconcentration performed before cardiopulmonary withdrawal is thought to be effective for hemostatic coagulation, such as ultrafiltration.

We have mentioned the unmeasured confounding bias in the manuscript, as follows.

“First, we cannot rule out the effect of unmeasured confounding bias, including the amount of blood loss and more detailed CPB information, which may skew our results; nevertheless, we used a multilevel propensity score analysis with good balance and examined significance using the McNemar test.” (lines 248-251)
